# Does the oviparity-viviparity transition alter the partitioning of yolk in embryonic snakes?

**DOI:** 10.1186/s12862-017-1083-z

**Published:** 2017-11-29

**Authors:** Yan-Qing Wu, Yan-Fu Qu, Xue-Ji Wang, Jian-Fang Gao, Xiang Ji

**Affiliations:** 1 Jiangsu Key Laboratory for Biodiversity and Biotechnology, College of Life Sciences, Nanjing Normal University, Nanjing, Jiangsu 210023 China; 2 Hangzhou Key Laboratory for Animal Adaptation and Evolution, College of Life and Environmental Sciences, Hangzhou Normal University, Hangzhou, Zhejiang 310036 China

**Keywords:** Offspring, Oviparity, Parity mode, Residual yolk, Snake, Viviparity, Yolk partitioning

## Abstract

**Background:**

The oviparity-viviparity transition is a major evolutionary event, likely altering the reproductive process of the organisms involved. Residual yolk, a portion of yolk remaining unutilized at hatching or birth as parental investment in care, has been investigated in many oviparous amniotes but remained largely unknown in viviparous species. Here, we used data from 20 (12 oviparous and 8 viviparous) species of snakes to see if the oviparity-viviparity transition alters the partitioning of yolk in embryonic snakes. We used ANCOVA to test whether offspring size, mass and components at hatching or birth differed between the sexes in each species. We used both ordinary least squares and phylogenetic generalized least squares regressions to test whether relationships between selected pairs of offspring components were significant. We used phylogenetic ANOVA to test whether offspring components differed between oviparous and viviparous species and, more specifically, the hypothesis that viviparous snakes invest more in the yolk as parental investment in embryogenesis to produce more well developed offspring that are larger in linear size.

**Results:**

In none of the 20 species was sex a significant source of variation in any offspring component examined. Newborn viviparous snakes on average contained proportionally more water and, after accounting for body dry mass, had larger carcasses but smaller residual yolks than did newly hatched oviparous snakes. The rates at which carcass dry mass (CDM) and fat body dry mass (FDM) increased with residual yolk dry mass (YDM) did not differ between newborn oviparous and viviparous snakes. Neither CDM nor FDM differed between newborn oviparous and viviparous snakes after accounting for YDM.

**Conclusions:**

Our results are not consistent with the hypothesis that the partitioning of yolk between embryonic and post-embryonic stages differs between snakes that differ in parity mode, but instead show that the partitioning of yolk in embryonic snakes is species-specific or phylogenetically related. We conclude that the oviparity-viviparity transition does not alter yolk partitioning in embryonic snakes.

**Electronic supplementary material:**

The online version of this article (10.1186/s12862-017-1083-z) contains supplementary material, which is available to authorized users.

## Background

It is widespread and perhaps ubiquitous among invertebrates and non-mammalian vertebrates that embryos complete development without depleting the entire yolk reserve [1–5]. The yolk remaining unutilized at hatching (oviparous species) or birth (viviparous species), namely residual yolk, is internalized into the abdominal cavity of the offspring before emergence from the egg or mother. This portion of yolk can be subsequently metabolized during the first days, weeks, or even months of life as a source of energy for maintenance metabolism and other essential activities prior to successful foraging [6–10] and contribute directly or indirectly to somatic tissue growth and thus linear growth ([11–13]; but see also [14–16]).

From previous studies on a wide range of vertebrate taxa we know the following. First, the size of residual yolk varies among species or taxa, among populations of the same species, among clutches of the same population or family, and even between the sexes of the same clutch [5, 9, 17–19]. For example, lizards (0−12% of the body dry mass, with a mean of 5%) generally have smaller residual yolks than do other reptiles (5−42% of the body dry mass, with a mean of 21%) and birds (21−56% of the body dry mass, with a mean of 34%) studied so far (Additional file 1: Table S1). Second, embryos cannot decide whether or not to use up the yolk or save some for later use, although residual yolk has a function to buffer the embryos from unpredictable environments and its quantity is affected by the environmental conditions experienced during embryonic development [20–22]. For example, high incubation or gestation temperatures and/or dry substrates often result in less developed offspring that characteristically have larger residual yolks but are smaller in linear size [23–27]. Third, fat-rich residual yolks better support post-hatch or post-natal activity or maintenance metabolism, whereas protein-rich residual yolks better support post-hatch growth [22, 28, 29]. Fourth, residual yolks are especially important for species where neonates and hatchlings have substantial energy expenditure before they begin to feed, including digging out of subterranean nests, long distance dispersal, or overwintering in nest cavities ([7, 9, 21, 30]; but see also [16, 31]). Fifth, residual yolks seem to be essential in species that have less developed feeding appendages, organs, behaviors, gut flora and/or enzyme systems at hatching or birth [2, 10, 32–34].

While residual yolk has been examined in a number of oviparous species, the occurrence, size and functional role of residual yolk in viviparous species remain almost unknown in non-mammalian amniote vertebrates. Oviparity is an ancestral mode of reproduction from which viviparity evolved independently ([35, 36]; but see also [37]). Viviparity has evolved in at least 115 lineages of squamate reptiles (lizards, snakes and amphisbaenians), and about one fifth of squamate reptiles are viviparous [38]. To our knowledge, however, the short-tailed pit-viper (*Gloydius brevicaudus*) is the only viviparous reptile for which the ratio of residual yolk dry mass to body dry mass has been reported [25]. The observation that the ratio and thus the relative size of residual yolk is far smaller in *G. brevicaudus* (3% of the body dry mass [25]) than in any oviparous snake (15−32% of the body dry mass, with an overall mean of 23%; Additional file 1: Table S1) studied so far raises a question that forms the basis of this study: Does the evolutionary transition from oviparity to viviparity alter yolk partitioning in embryonic snakes? If so, one may hypothesize that the partitioning of yolk between embryonic (parental investment in embryogenesis, PIE) and post-embryonic (parental investment in care, PIC) stages should differ between oviparous and viviparous species, and in particular, viviparous species should produce more fully developed offspring that are larger in linear body size but have smaller residual yolks. Alternatively, it is possible that a smaller amount of yolk remaining at birth simply results from less yolk invested by viviparous species as PIC, perhaps owing to the increased maternal survival costs during gestation associated with carrying the yolk exceeding the need to produce a complete offspring [39, 40]. In the latter case, neonates of viviparous species should have smaller residual yolks when compared to newly hatched hatchlings of oviparous species of the same developmental condition.

Snakes are an ideal taxon for studying whether the developmental condition and yolk partitioning strategies are associated with parity mode because their viviparous species do not belong to any lineage that exhibits placentotrophy but rather all are lecithotrophic and yolk reserves support all energy demands during embryogenesis [41, 42]. Lecithotrophic viviparous species actually are similar to oviparous species in egg yolk and embryonic development and nutritional pattern [43]. Here, we used data collected from 20 (12 oviparous and 8 viviparous) species of snakes to address the above question.

## Methods

Snakes were collected from three provinces in mainland China between 1998 and 2015, with four species from Guangxi in South China, 14 from Zhejiang in East China and two from Liaoning in Northeast China (Table 1). Detailed procedures for maintenance of gravid females and collection of eggs and newborn offspring, hatchlings (oviparous species) and neonates (viviparous species), have been described elsewhere [25, 44–47]. In brief, wild-caught gravid females were brought to our laboratory, where 1−3 females were housed in each wire (for terrestrial species) or glass (for aquatic species) cage until they laid eggs or gave birth to young. Cages were placed in an indoor animal holding facility where temperatures never varied outside the range of 24−30 °C. Food [oriental weatherfish (*Misgurnus anguillicaudatus*), common toads (*Bufo gargarizans*), rice frogs (*Fejervarya limnocharis*), black-spotted frogs (*Pelophylax nigromaculata*), or house mice (*Mus musculus*)] and water were provided ad libitum. Eggs were collected and weighed less than 3 h post-laying. Eggs were either dissected to identify Zehr’s [48] embryonic stage or incubated under multiple thermal conditions using Binder KB (Binder, Germany) or Shellab (Sheldon MFG Inc., USA) incubators. Hatchlings or neonates were collected, weighed, measured for snout-vent length (SVL) and tail length, and sexed (by manual eversion of hemipenes) less than 6 h emergence from the egg or mother. As extreme incubation temperatures often result in hatchlings that have either smaller (low temperatures) or larger (high temperatures) than usual residual yolks, only hatchlings from eggs incubated at temperatures moderate for each species were used in this study.

**Table 1:** Descriptive statistics, expressed as mean ± SE and range, for size, mass and three main body components of newly hatched (oviparous species) and newborn (viviparous species) snakes

Species	Parity mode	*N* (M/F)	SVL (mm)	Tail length (mm)		Wet body mass (g)	Dry body mass (g)	Carcass dry mass (g)	Fat body dry mass (g)	Residual yolk dry mass (g)
				Males	Females					
*Bungarus multicinctus* ^1^	O	20/21	242.9 ± 2.4 205−269	40.7 ± 0.7 33−46	38.3 ± 0.4 34−43	6.8 ± 0.2 3.9−8.8	1.82 ± 0.06 0.83−2.43	1.00 ± 0.03 0.62−1.30	0.24 ± 0.01 0.12−0.39	0.57 ± 0.03 0.08−0.96
*Coelognathus radiatus* ^1^	O	16/14	303.3 ± 3.1 277−339	72.1 ± 1.0 66−78	70.8 ± 1.3 62−79	11.1 ± 0.2 8.7−13.2	2.96 ± 0.06 2.28−3.49	2.12 ± 0.04 1.72−2.49	0.49 ± 0.02 0.32−0.67	0.34 ± 0.02 0.19−0.71
*Deinagkistrodon acutus* ^2^	O	16/16	242.7 ± 2.1 215−268	51.0 ± 0.7 47−55	46.2 ± 0.7 42−51	14.2 ± 0.2 11.6−16.6	3.71 ± 0.07 2.68−4.36	1.88 ± 0.04 1.53−2.39	0.56 ± 0.02 0.37−0.77	1.27 ± 0.06 0.26−1.88
*Dinodon rufozonatum* ^2^	O	15/19	206.4 ± 2.4 176−238	48.7 ± 1.7 36−58	48.4 ± 0.9 41−56	4.4 ± 0.2 3.3−6.0	1.24 ± 0.05 0.85−1.81	0.80 ± 0.03 0.55−1.09	0.22 ± 0.01 0.09−0.34	0.22 ± 0.02 0.09−0.48
*Elaphe carinata* ^2^	O	41/45	376.9 ± 2.5 321−432	89.0 ± 1.5 64−109	85.9 ± 1.0 74−100	23.7 ± 0.6 14.3−36.3	6.38 ± 0.19 3.44−10.19	3.62 ± 0.09 2.25−5.96	1.05 ± 0.04 0.41−2.01	1.71 ± 0.07 0.67−3.92
*Elaphe taeniura* ^1^	O	29/35	359.9 ± 3.1 303−446	92.9 ± 1.6 75−116	92.9 ± 1.3 75−112	16.8 ± 0.3 12.1−22.1	4.89 ± 0.10 3.47−7.08	2.91 ± 0.10 1.55−4.48	0.88 ± 0.03 0.30−1.54	1.10 ± 0.08 0.26−2.62
*Naja atra* ^2^	O	28/30	269.4 ± 1.5 241−293	50.1 ± 0.7 40−58	45.9 ± 0.5 40−53	12.6 ± 0.3 8.6−17.9	3.08 ± 0.11 1.79−5.17	1.75 ± 0.04 1.15−2.48	0.49 ± 0.02 0.21−0.82	0.84 ± 0.06 0.31−2.29
*Ptyas korros* ^2^	O	43/41	226.8 ± 1.5 188−255	93.9 ± 0.8 82−106	94.6 ± 1.3 68−108	6.7 ± 0.1 4.6−8.4	1.73 ± 0.03 1.12−2.32	1.26 ± 0.02 0.88−1.62	0.24 ± 0.01 0.12−0.42	0.24 ± 0.01 0.06−0.55
*Ptyas mucosus* ^2^	O	7/8	335.4 ± 2.9 310−354	101.6 ± 1.8 94−109	102.2 ± 2.2 93−111	16.2 ± 0.5 12.3−19.2	4.19 ± 0.16 2.84−5.50	2.85 ± 0.09 2.16−3.36	0.70 ± 0.05 0.37−1.16	0.63 ± 0.05 0.31−0.97
*Rhabdophis tigrinus lateralis* ^2^	O	17/17	148.9 ± 1.4 126−163	37.7 ± 0.8 33−44	35.0 ± 0.8 24−40	2.2 ± 0.05 1.3−2.8	0.52 ± 0.01 0.36−0.65	0.35 ± 0.01 0.26−0.45	0.04 ± 0.002 0.02−0.08	0.12 ± 0.007 0.04−0.20
*Xenochrophis piscator* ^1^	O	27/26	130.8 ± 0.8 117−144	48.5 ± 0.8 37−53	42.9 ± 0.5 34−46	1.6 ± 0.02 1.3−1.9	0.37 ± 0.004 0.32−0.43	0.28 ± 0.003 0.22−0.32	0.04 ± 0.001 0.02−0.07	0.04 ± 0.002 0.01−0.11
*Zaocys dhumnades* ^2^	O	14/16	288.5 ± 3.0 244−317	101.9 ± 1.8 91−117	100.3 ± 2.0 84−112	8.2 ± 0.2 5.7−10.3	2.23 ± 0.06 1.57−2.75	1.61 ± 0.04 1.04−2.10	0.34 ± 0.02 0.13−0.53	0.29 ± 0.02 0.11−0.52
*Elaphe rufodorsata* ^2^	V	12/12	171.2 ± 2.0 153−194	38.3 ± 0.8 35−43	33.4 ± 0.6 30−37	3.0 ± 0.08 2.1−3.5	0.69 ± 0.02 0.46−0.82	0.57 ± 0.01 0.40−0.70	0.10 ± 0.005 0.04−0.13	0.02 ± 0.004 0.001−0.07
*Enhydris chinensis* ^2^	V	10/10	150.9 ± 2.0 140−174	30.4 ± 0.5 28−34	25.8 ± 0.6 24−30	3.2 ± 0.1 2.7−4.7	0.70 ± 0.03 0.52−1.12	0.58 ± 0.03 0.45−0.96	0.11 ± 0.007 0.06−0.16	0.007 ± 0.002 0.001−0.027
*Enhydris plumbea* ^2^	V	13/14	119.9 ± 1.7 104−154	21.5 ± 0.3 20−23	19.0 ± 0.3 16−21	1.5 ± 0.05 1.2−2.0	0.34 ± 0.01 0.20−0.51	0.31 ± 0.01 0.20−0.43	0.03 ± 0.003 0.005−0.63	0.004 ± 0.001 0.001−0.014
*Gloydius brevicaudus* ^2^	V	12/12	173.9 ± 1.4 162−189	29.7 ± 0.5 27−32	26.4 ± 0.3 25−28	3.7 ± 0.1 3.0−4.8	0.79 ± 0.02 0.64−1.02	0.69 ± 0.01 0.58−0.88	0.08 ± 0.003 0.04−0.11	0.02 ± 0.002 0.01−0.05
*Gloydius saxatilis* ^3^	V	12/14	220.5 ± 2.6 187−240	29.1 ± 0.9 24−36	28.5 ± 0.7 24−33	5.4 ± 0.2 3.5−8.1	1.16 ± 0.04 0.75−1.75	0.81 ± 0.03 0.53−1.25	0.31 ± 0.02 0.04−0.64	0.04 ± 0.01 0.001−0.24
*Gloydius ussurensis* ^3^	V	13/10	214.2 ± 2.9 186−240	32.6 ± 1.1 26−41	32.4 ± 1.0 27−37	4.7 ± 0.2 3.0−6.5	1.00 ± 0.05 0.65−1.39	0.73 ± 0.03 0.51−1.00	0.24 ± 0.02 0.10−0.54	0.03 ± 0.006 0.001−0.10
*Macropisthodon rudis* ^2^	V	13/18	157.9 ± 1.1 145−172	38.7 ± 0.6 35−42	32.3 ± 0.6 28−38	4.0 ± 0.06 3.0−4.7	0.86 ± 0.02 0.63−1.04	0.69 ± 0.01 0.53−0.82	0.11 ± 0.006 0.05−0.17	0.07 ± 0.004 0.03−0.11
*Sinonatrix annularis* ^2^	V	13/13	157.9 ± 1.9 142−177	43.9 ± 0.6 40−47	41.0 ± 0.6 37−43	4.3 ± 0.1 2.9−5.7	1.19 ± 0.04 0.75−1.70	0.79 ± 0.03 0.54−1.12	0.20 ± 0.01 0.12−0.32	0.20 ± 0.01 0.08−0.30

Species with the same superscript were collected from the same province. ^1^: Guangxi (South China); ^2^: Zhejiang (East China); ^3^: Liaoning (Northeast China). O: oviparous species; V: viviparous species

A total of 762 newborn offspring, 15−86 hatchlings (one or two individuals of different sexes from each clutch) from each oviparous species and 20−31 neonates (one or two individuals of different sexes from each litter) from each viviparous species were euthanized by freezing at −20 °C on the day of hatching or birth. Frozen hatchlings and neonates were later thawed, dissected and separated into residual yolk, fat bodies and carcass. Freezing and thawing did not affect our ability to separate these components. The three components were dried to a constant mass in a 60 °C oven (Shanghai Senxin Ltd., China) for 48 h to obtain dry mass.

**Fig. 1:**
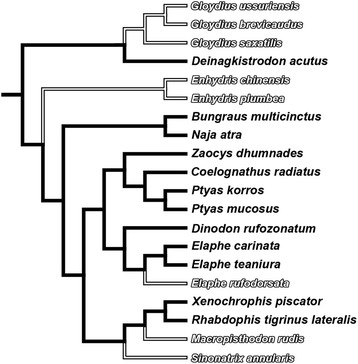
The phylogeny of the 20 species of snakes used in this study. The topology was inferred from the proximate phylogenetic relationships at the species-level [55–58] and drawn using Mesquite 3.04 [54]. Oviparous species are in solid font, and viviparous species in hollow font

We used one-way ANCOVA with wet body mass or SVL (see below for note) as the covariate to test whether offspring size, mass and components (carcass, residual yolk and fat bodies) at hatching or birth differed between the sexes in each species. The same analysis was also used to test the parallelism of regression lines between oviparous and viviparous species. We used phylogenetic ANOVA to test whether proportional amounts of water, dry carcass, dry fat bodies and dry residual yolk differed between oviparous and viviparous species. Proportional data were arc-sine transformed prior to further analyses. We calculated residuals of carcass dry mass (CDM = hatchling dry mass − fat body dry mass − yolk dry mass) and fat body dry mass (FDM) against residual yolk dry mass (YDM) for each species, and then analyzed them using phylogenetic ANOVA to test whether CDM and FDM differed between oviparous and viviparous species with the same amount of YDM in R 3.3.0 [49] with the package GEIGER [50]. We used ordinary least squares (OLS) and phylogenetic generalized least squares (PGLS) regressions in R 3.3.0 with the packages RMS [51] and CAPER [52] to test whether relationships between selected pairs of offspring components were significant. The Akaike’s Information Criterion (AIC) and likelihood-ratio test [53] were used to assess the adequacy of models tested. We performed phylogenetic ANOVA and PGLS to account for the non-independence of data due to the shared evolutionary history of species. To do that, we reconstructed a phylogeny for the 20 species using Mesquite 3.04 [54] based on the species-level phylogenetic relationships proposed by Pyron et al. [55, 56] and others [57, 58] for species of the families Colubridae, Elapidae, Natricinae and Viperidae (Fig. 1). We could not estimate individual branch lengths because sequences for reconstructing the phylogeny were either unavailable (*Bungarus multicinctus* and *Gloydius ussurensis*) or incomplete for some species (e.g. *Coelognathus radiatus* and *Macropisthodon rudis*). Consequently, we arbitrarily set branch lengths to 1 (Fig. 1), which is appropriate for a speciation model of evolution [59]. Phylogenetic signal was measured by Pagel’s lambda (λ) [60], which indicates the strength of the phylogenetic relationship. Lambda values of or near 0 indicate phylogenetic independence; values of or near 1 indicate that the variable is fully explained by evolutionary history and thus shows the maximal strength of phylogenetic signal [60]. Throughout this paper, values are presented as mean ± SE and range, and the significance level is set at *P* = 0.05.

## Results

Descriptive statistics for body size, mass and components of newborn offspring are given in Table 1. Two oviparous species (*Deinagkistrodon acutus* and *Xenochrophis piscator*) showed sexual size dimorphism at hatching, and in both species females were longer in SVL after accounting for wet body mass (ANCOVA: both *P* < 0.004). Six oviparous (*B. multicinctus*, *D. acutus*, *Elaphe carinata*, *Naja atra*, *Rhabdophis tigrinus lateralis* and *X. piscator*) and six viviparous (*Elaphe rufodorsata*, *Enhydris chinensis*, *Enhydris plumbea*, *Gloydius brevicaudus*, *M. rudis* and *Sinonatrix annularis*) species showed sexual dimorphism in tail length at hatching or birth, and in all these species males were longer in tail length after accounting for SVL (ANCOVA: all *P* < 0.01). In none of the 20 species did we find that total body dry mass, CDM, FDM, or YDM differed between the sexes after accounting for wet body mass (ANCOVA: all *P* > 0.093).

**Fig. 2:**
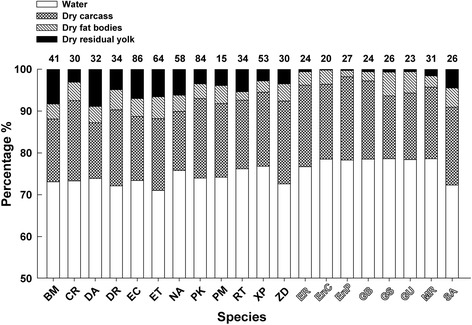
Percentages of four major body components in newly hatched or newborn snakes. Numbers in the figure are sample sizes. Solid abbreviations represent oviparous species, and hollow abbreviations represent viviparous species. BM: *B. multicinctus*; CR: *C. radiatus*; DA: *D. acutus*; DR: *D. rufozonatum*; EC: *E. carinata*; ET: *E. taeniura*; NA: *N. atra*; PK: *P. korros*; PM: *P. mucosus*; RT: *R. T. lateralis*; XP: *X. piscator*; ZD: *Z. dhumnades*; ER: *E. rufodorsata*; EnC: *En. chinensis*; EnP: *En. plumbea*; GB: *G. brevicaudus*; GS: *G. saxatilis*; GU: *G. ussurensis*; MR: *M. rudis*; and SA: *S. annularis*

In the oviparous taxon species mean values for hatchling water contents ranged from 71% (*Elaphe taeniura*) to 77% (*X. piscator*) of body wet mass, with a mean of 74%; in the viviparous taxa species mean values for neonate water contents ranged from 72% (*S. annularis*) to 79% (*En. chinensis*, *G. brevicaudus*, *G. saxatilis* and *M. rudis*) of body wet mass, with a mean of 78% (Fig. 2). Newborn viviparous snakes on average contained proportionally more water than did newly hatched oviparous snakes (phylogenetic ANOVA: *F*_1, 18_ = 17.28, *P* < 0.001). In the oviparous taxon species mean proportions of CDM to hatchling dry mass ranged from 51% (*D. acutus*) to 76% (*X. piscator*), with a mean of 65%; in the viviparous taxa species mean proportions of CDM to neonate dry mass ranged from 67% (*S. annularis*) to 92% (*En. plumbea*), with a mean of 80% (Fig. 2). Newborn viviparous snakes on average had relatively larger carcasses than did newly hatched oviparous snakes (phylogenetic ANOVA; *F*_1, 18_ = 8.99, *P* < 0.01). Species mean proportions of FDM to hatchling dry mass ranged from 9% (*R. T. lateralis*) to 18% (*E. taeniura*), with a mean of 15%; species mean proportions of FDM to neonate dry mass ranged from 7% (*En. plumbea*) to 27% (*G. saxatilis*), with a mean of 16% (Fig. 2). Newborn viviparous snakes did not differ from newly hatched oviparous snakes in the relative size of fat bodies (phylogenetic ANOVA: *F*_1, 18_ = 0.25, *P* = 0.630). Species mean proportions of YDM to hatchling dry mass ranged from 12% (*C. radiatus*) to 34% (*D. acutus*), with a mean of 20%; species mean proportions of YDM to neonate dry mass ranged from 1% (*En. chinensis* and *En. plumbea*) to 16% (*S. annularis*), with a mean of 5% (Fig. 2). Newborn viviparous snakes on average had relatively smaller residual yolks than did newly hatched oviparous snakes (phylogenetic ANOVA: *F*_1, 18_ = 25.29, *P* < 0.0001).

**Fig. 3:**
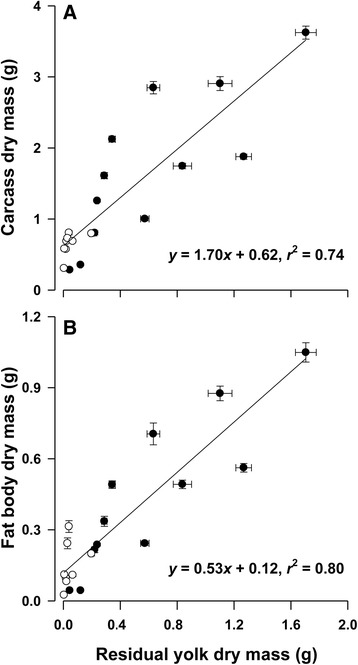
Carcass dry mass (**a**) and fatbody dry mass (**b**) in relation to residual yolk dry mass. Data are expressed as mean ± SE. Regression equations and coefficients are given in the figure. Solid dots represent oviparous species, and open dots represent viviparous species

Regression lines of CDM against YDM for oviparous and viviparous species were parallel (ANCOVA: *F*_1, 16_ = 0.006, *P* = 0.938), and so were regression lines of FDM against YDM (ANCOVA: *F*_1, 16_ = 0.003, *P* = 0.956). CDM (phylogenetic ANOVA: *F*_1, 18_ = 0.83, *P* = 0.375) and FDM (phylogenetic ANOVA: *F*_1, 18_ = 0.34, *P* = 0.569) did not differ between oviparous and viviparous species after accounting for YDM. Data pooled for oviparous and viviparous species showed that YDM explained 74% of variation in CDM, and 80% of variation in FDM (Fig. 3). The three offspring body components were positively related to each other, with all these relationships exhibiting strong phylogenetic signals (Table 2).

**Table 2:** Parameters of regressions between each pair of three main body components (dry carcass, dry residual yolk and dry fat bodies) estimated with ordinary least squares (OLS) and phylogenetic generalized least squares (PGLS) regression models

Models	*N*	Slope	Elevation	*r* ^2^	ln likelihood	AIC	λ	*F*_1, 18_	*P-*value
OLS regression model									
Carcass vs Residual yolk	20	1.70 ± 0.23	0.62 ± 0.14	0.74	−13.43	32.86		52.34	< 0.0001
Fat bodies vs Residual yolk	20	0.53 ± 0.06	0.12 ± 0.04	0.80	13.27	−20.55		73.84	< 0.0001
Carcass vs Fat bodies	20	3.25 ± 0.16	0.23 ± 0.07	0.96	4.16	−2.33		390.63	< 0.0001
PGLS regression model									
Carcass vs Residual yolk	20	1.50 ± 0.19	0.39 ± 0.31	0.76	−8.50^a^	25.00	0.84	59.90	< 0.0001
Fat bodies vs Residual yolk	20	0.46 ± 0.06	0.10 ± 0.07	0.79	15.01	−22.03	0.54	59.52	< 0.0001
Carcass vs Fat bodies	20	3.13 ± 0.17	0.16 ± 0.13	0.95	7.26^a^	−6.53	0.75	353.10	< 0.0001

Models with a superscript of ^a^ are significantly better than their alternate OLS or PGLS models

## Discussion

While 12 species showed sexual dimorphism in SVL and/or tail length at hatching or birth, in none of the 20 species was sex a significant source of variation in the size of carcass, residual yolk, or fat bodies. This suggests that offspring sex is not related to maternal allocation of resources into egg yolk or the partitioning of yolk between PIE and PIC in snakes. Reptiles of different species or taxa show similar patterns of embryonic growth or yolk depletion in the course of embryonic development, which generally include three phases. The first phase is one of minimal transfer of energy and material from yolk to embryo, and the second and third phases are characterized by accelerated and, after an inflexion, decelerated embryonic growth or yolk depletion [61–65]. However, as we observed in this study (Fig. 2), yolk allocation strategies and thus proportions of yolk allocated to produce either larger offspring with smaller yolk reserves or smaller offspring with larger amounts of residual yolk may vary considerably among species [10, 66–69]. Natural selection for a given level of PIC is essentially influenced by the feeding ability of newborns or the period when they have a negative energy balance [7, 9, 21, 30]. Thus, while larger residual yolks would provide sustenance for longer periods and better support early growth, larger offspring with smaller yolk reserves could be favored when resources are abundant or selection for high performance is strong [21, 22, 69, 70].

The first 1−3 weeks of life for oviparous snakes represent a period of time when they do not eat but often become even more fully developed as the consequence of early growth achieved by the post-hatching transfer of energy and material from residual yolk to carcass [44, 66–68]. Previous studies on several species of oviparous snakes including *Dinodon rufozonatum* [71], *E. carinata* [67], *E. taeniura* [68] and *Ptyas korros* [66] consistently show that more fully developed hatchlings are longer, have smaller residual yolks, and hold more water largely due to metabolic water production accompanied by yolk depletion. Here, we found that newborn viviparous snakes on average contained proportionally more water and, after accounting for body dry mass, they had larger carcasses but smaller residual yolks than did newly hatched oviparous snakes (Fig. 2). These findings suggest that viviparous snakes generally produce more fully developed offspring than oviparous snakes, but they do not support the hypothesis that the partitioning of yolk between PIE and PIC differs between snakes with different parity modes for two reasons. First, the partitioning of yolk between PIE and PIC and the degree of development at hatching or birth vary considerably among species in both oviparous and viviparous snakes (Fig. 2). This suggests that yolk partitioning in embryonic snakes is unlikely to be associated with parity mode but rather to be species-specific or phylogenetically related. In four aquatic viviparous snakes, for example, the relative size of carcass at birth was much smaller in *S. annularis* than in the other three species (*E. rufodorsata*, *En. chinensis* and *En. plumbea*; 67% versus 83−91% of the body dry mass), whereas the reverse occurred for the relative size of residual yolk (16% versus 1−3% of the body dry mass; Fig. 2). Second, viviparous snakes do not always produce more fully developed offspring with larger carcasses and smaller residual yolks. *Sinonatrix annularis* also offers an example, as its relative size of carcass was smaller than a half of 12 oviparous species studied herein, including *C. radiatus*, *P. korros*, *Ptyas mucosus*, *R. T. lateralis*, *X. piscator* and *Zaocys dhumnades* (67% versus 68−76% of the body dry mass; Fig. 2).

Given equal offspring mass, residual yolk mass is inversely related to yolk-free offspring mass ([61–65, 72]; but see also [22]). Here, we found in both oviparous and viviparous snakes that greater residual yolk mass occurred in species that produced larger offspring with larger carcasses and fat bodies and thus were heavier in yolk-free mass (Fig. 3). We also found that oviparous and viviparous snakes displayed the same rates at which CDM and FDM increased with YDM (Fig. 3) and that neither CDM nor FDM differed between newborn oviparous and viviparous snakes after accounting for YDM. These findings, when coupled with strong phylogenetic signals in all relationships between selected pairs of body components, allow us to conclude that the partitioning of yolk in embryonic snakes and the level of residual yolks or PIC are not associated with parity mode, but are instead species-specific or phylogenetically related.

Why do some snakes produce more fully developed offspring that emerge from the egg or mother later whereas others do not? To answer this question, we need to make a cost-benefit assessment. Snakes emerging earlier from the egg or mother have shorter lengths of embryonic development and larger residual yolks. For example, the mean incubation length at any temperature across the range within which embryonic development can take place is shorter in *E. carinata* (YDM accounting for 27% of total dry mass) than in *E. taeniura* (YDM accounting for 22% of total dry mass) [73, 74]. The benefit to an individual emerging immediately after completion of embryonic development is to initiate locomotion, feeding and growth toward maturity as soon as possible. This benefit is especially important for oviparous reptiles where parental care, if present, is quite limited [75, 76]. The benefit of delayed emergence from the egg or mother is the proven sanctuary offered during a period of time when the benefit mentioned above is likely to be outweighed by predation or by mortality resulting from unfavorable environmental conditions [77, 78]. Potential costs of remaining in the egg or mother after completion of embryonic development is the increased mortality as a result of prolonged exposure of eggs or mothers to predators and other hostile factors, whereas potential costs of immediate emergence result primarily from high probabilities that newborns will encounter unfavorable situations such as earlier exposure to predators, drying up aquatic habitats or the onset of winter. These risks, when coupled with the lack of feeding ability in the first days or weeks of life, would result in immediate emergence being disadvantageous unless the disadvantages of remaining in the nest or mother were even higher. Delayed emergence is recognized as an adaptive trait for freshwater turtles where young individuals leaving the nest at the time of hatching might encounter inhospitable environmental conditions [77–79]. Unfortunately, parallel work on snakes has yet to be conducted. However, if delayed emergence is also adaptive for snakes, we predict that it will be most likely to occur in species that live in unpredictable and inhospitable environments with high levels of uncertainty about the cost-benefit of emergence. Future work could usefully investigate more lineages of reptiles with both oviparous and viviparous species also in a phylogenetic context to test this prediction.

## Conclusions

Oviparity and viviparity are two distinct modes of reproduction that entail both advantages and disadvantages, of which each may differ in their applicability to particular species. Advantages and disadvantages associated with oviparity cannot be found in viviparity, and vice versa. However, oviparous and viviparous reptiles are common in that prolonged embryonic development increases not only energetic costs but also survival costs due to prolonged exposure of eggs or pregnant females to hostile biotic (such as predators and pathogenic microbes) and abiotic (such as lethal thermal and/or hydric extremes) factors. Our results are not consistent with the hypothesis that viviparous snakes have smaller amounts of residual yolk because they invest more in the yolk as PIE to produce more well developed offspring that are larger in linear size. Also, our results are not consistent with an alternative possibility that viviparous snakes have smaller amounts of residual yolk because they invest less in the yolk as PIC to reduce survival costs during gestation associated with carrying the yolk exceeding the need to produce a complete offspring. Instead, our results show that the partitioning of yolk in embryonic snakes is species-specific or phylogenetically related, thus allowing us to conclude that the oviparity-viviparity transition does not alter yolk partitioning in embryonic snakes.

## Additional file

Additional file 1: Table S1. Non-mammalian amniotes for which data on the ratio of residual yolk dry mass to total hatchling dry mass have been available. O: oviparity; V: viviparity. (DOC 155 kb)

## Abbreviations

CDM: Carcass dry mass (= hatchling dry mass − fat body dry mass − yolk dry mass)
FDM: Fat body dry mass
PIC: Parental investment in care
PIE: Parental investment in embryogenesis
SVL: Snout-vent length
YDM: Yolk dry mass
