# Supplementary material for: Does the oviparity-viviparity transition alter the partitioning of yolk in embryonic snakes?
Source: BMC Evol Biol. 2017 Nov 29;17:235. doi: 10.1186/s12862-017-1083-z (PMC5707827; doi:10.1186/s12862-017-1083-z)
Supplement: Additional file 1: Table S1. — Non-mammalian amniotes for which data on the ratio of residual yolk dry mass to total hatchling dry mass have been available. O: oviparity; V: viviparity. (DOC 155 kb) [file 12862_2017_1083_MOESM1_ESM.doc]

**Table S1** Non-mammalian amniotes for which data on the ratio of residual yolk dry mass to total hatchling dry mass have been available. O: oviparity; V: viviparity

| Taxon | Species | Parity mode | % | Reference |
| --- | --- | --- | --- | --- |
| Turtles | *Apalone mutica* | O | 29 | [1] |
|  | *Caretta caretta* | O | 12 | [2] |
|  | *Chelodina expansa* | O | 5 | [3] |
|  | *Chelydra serpentina* | O | 24 | [4] |
|  | *Chrysemys picta* | O | 26 | [5] |
|  | *Emydura signata* | O | 6 | [6] |
|  | *Pelodiscus sinensis* | O | 7 | [7] |
|  | *Trachemys scripta elegans* | O | 26 | [8] |
|  | *Trachemys scripta elegans* | O | 25 | [9] |
| Lizards | *Amphibolurus barbatus* | O | 1 | [10] |
|  | *Calotes versicolor* | O | 10 | [11] |
|  | *Eremias argus* | O | 5 | [12] |
|  | *Eremias brenchleyi* | O | 6 | [13] |
|  | *Gekko japonicus* | O | 3 | [14] |
|  | *Lampropholis delicata* | O | 0 | [15] |
|  | *Lampropholis guichenoti* | O | 0 | [15] |
|  | *Plestiodon chinensis* | O | 12 | [16] |
|  | *Plestiodon chinensis* | O | 10 | [17] |
|  | *Podarcis muralis* | O | 1 | [18] |
|  | *Takydromus wolteri* | O | 3 | [19] |
| Snakes | *Bungarus multicintus* | O | 30 | [20] |
|  | *Deinagkistrodon acutus* | O | 32 | [21] |
|  | *Dinodon rufozonatum* | O | 19 | [22] |
|  | *Elaphe carinata* | O | 25 | [23] |
|  | *Elaphe taeniura* | O | 15 | [24] |
|  | *Gloydius brevicaudus* | V | 3 | [25] |
|  | *Naja atra* | O | 25 | [26] |
|  | *Ptyas korros* | O | 17 | [27] |
|  | *Ptyas mucosus* | O | 16 | [28] |
|  | *Rhabdophis tigrinus lateralis* | O | 25 | [29] |
| Crocodiles | *Alligator mississippiensis* | O | 42 | [30] |
|  | *Alligator mississippiensis* | O | 41 | [31] |
| Birds | *Alectura lathami* | O | 21 | [32] |
|  | *Anas platyrhynchos* | O | 21 | [33] |
|  | *Gallus gallus domesticus* | O | 39 | [34] |
|  | *Struthio camelus* | O | 56 | [35] |

**References**

1. Nagle RD, Plummer MV, Congdon JD, Fischer FU. Parental investment, embryo growth, and hatchling lipid reserves in softshell turtles (*Apalone mutica*) from Arkansas. Herpetologica. 2003;59:145−154.
2. Kraemer JE, Bennett SH. Utilization of posthatching yolk in loggerhead sea turtles, *Caretta caretta*. Copeia. 1981;1981:406–411.
3. Booth DT. Incubation of rigid-shelled turtle eggs: Do hydric conditions matter? J Comp Physiol B. 2002;172:627–33.
4. Packard GC, Packard MJ, Miller K, Boardman TJ. Influence of moisture, temperature, and substrate on snapping turtle eggs and embryos. Ecology. 1987;68:983−993.
5. Gutzke WHN, Packard GC, Packard MJ, Boardman TJ. Influence of the hydric and thermal environments on eggs and hatchlings of painted turtles (*Chrysemys picta*). Herpetologica. 1987;43:393−404.
6. Booth, D. T., and C. Y. Yu. 2009. Influence of the hydric environment on water exchange and hatchlings of rigid-shelled turtle eggs. Physiological and Biochemical Zoology 82:382–387.
7. Du WG, Ji X, Xu WQ. Dynamics of material and energy during incubation in the soft-shelled turtle (*Pelodiscus sinensis*). Acta Zool Sin. 2001;47:371−375.
8. Filoramo NI, Janzen FJ. Effects of hydric conditions during incubation on overwintering hatchlings of the red-eared slider turtle (*Trachemys scripta elegans*). J Herpetol. 1999;33:29−35.
9. Tucker JK, Filoramo NI, Paukstis GL, Janzen FJ. Residual yolk in captive and wild-caught hatchlings of the red-eared slider turtle (*Trachemys scripta elegans*). Copeia. 1998;1998:488−492.
10. Packard MJ, Packard GC, Miller JD, Jones ME, Gutzke WHN. Calcium mobilization, water balance, and growth in embryos of the agamid lizard *Amphibolurus barbatus*. J Exp Zool. 1985;235:349−57.
11. Ji X, Qiu QB, Diong CH. Influence of incubation temperature on hatching success, embryonic use of energy, and size and morphology of hatchlings in the oriental garden lizard, *Calotes versicolor* (Agamidae). J Exp Zool. 2002;292:649−659.
12. Hao QL, Liu HX, Ji X. Phenotypic variation in hatchling Mongolian racerunners (*Eremias argus*) from eggs incubated at constant versus fluctuating temperatures. Acta Zool Sin. 2006;52:1049−1057.
13. Xu XF, Wu YL, Zhang JL. Influence of thermal and hydric environments on egg incubation, hatching success, and hatchling traits in a lacertid lizard, *Eremias brenchley*. Zool Res. 2005;26:55−60.
14. Xu XF, Ji X. Female reproduction and influence of incubation temperature on duration of incubation and hatchling traits in the gecko, *Gekko japonicu*s. Chin J Ecol. 2001;20(6):8−11.
15. Thompson MB, Speake BK, Russell KJ, McCartney RJ. Utilisation of lipids, protein, ions and energy during embryonic development of Australian oviparous skinks in the genus *Lampropholis*. Comp Biochem Physiol A. 2001;129:313−326.
16. Ji X, Zhang CH. Effects of thermal and hydric environments on incubating eggs, hatching success and hatchling traits in the Chinese skink (*Eumeces chinensis*). Acta Zool Sin. 2001;47:250−259.
17. Ji X, Huang HY, Hu XZ, Du WG. Geographic variation in reproductive characteristics and egg incubation of *Eumeces chinensis*. Chin J Appl Ecol. 2002;13:680−684.
18. Ji X, Braña F. The influence of thermal and hydric environments on incubating eggs and embryonic use of energy and nutrients in the wall lizard *Podarcis* *muralis*. Comp Biochem Physiol A. 1999;124:205−213.
19. Pan ZC, Ji X. The influence of incubation temperature on size, morphology, and locomotor performance of hatchling grass lizards (*Takydromus wolteri*). Acta Ecol Sin. 2001;21:2031−2038.
20. Ji X, Gao JF, Han J. Phenotypic responses of hatchlings to constant versus fluctuating incubation temperatures in the multi-banded krait, *Bungarus multicintus* (Elapidae). Zool Sci. 2007;24:384−390.
21. Lin ZH, Ji X, Luo LG, Ma XM. Incubation temperature affects hatching success, embryonic expenditure of energy and hatchling phenotypes of a prolonged egg-retaining snake, *Deinagkistrodon acutus* (Viperidae). J Therm Biol. 2005;30:289−297.
22. Ji X, Xu XF, Lin ZH. Influence of incubation temperature on characteristics of *Dinodon rufozonatum* (Reptilia: Colubridae) hatchlings, with comments on the function of residual yolk. Zool Res. 1999;20:342−346.
23. Ji X, Sun PY, Fu SY, Zhang HS. Utilization of energy and nutrients in incubating eggs and post-hatching yolk in a colubrid snake, *Elaphe* *carinata*. Herpetol J. 1997;7:7−12.
24. Du WG, Ji X. The effects of incubation temperature on hatching success, embryonic use of energy and hatchling morphology in the stripe-tailed ratsnake *Elaphe taeniura*. Asiat Herpetol Res. 2008;11:24−30.
25. Gao JF, Qu YF, Luo LG, Ji X. Evolution of reptilian viviparity: a test of the maternal manipulation hypothesis in a temperate snake, *Gloydius brevicaudus* (Viperidae). Zool Sci. 2010;27:248−255.
26. Ji X, Du WG. The effects of thermal and hydric conditions on incubating eggs and hatchling traits in the cobra, *Naja naja atra*. J Herpetol. 2001;35:186−194.
27. Du, W. G., and X. Ji. 2002. Effects of incubation temperature on duration of incubation, hatching success, and hatchling traits in the gray rat snake, *Ptyas korros* (Colubridae). Acta Ecologica Sinica 22:548−553.
28. Lin ZH, Ji X. Reproductive output and effects of incubation thermal environments on hatchling phenotypes of mucous ratsnake (*Ptyas mucous*). Acta Zool Sin. 2004;50:541−550.
29. Chen HL, Ji X. The effects of thermal environments on duration of incubation, hatching success and hatchling traits in a colubrid snake, *Rhabdophis tigrinus lateralis*. Acta Ecol Sin. 2002;22:1850−1858.
30. Congdon JD, Gibbons JW. Posthatching yolk reserves in hatchling American alligators. Herpetologica. 1989;45:305−309.
31. Fischer RU, Mazzotti FJ, Congdon JD, Gatten Jr RE. Post-hatching yolk reserves: parental investment in American alligators from Louisiana. J Herpetol. 1991;25:253−256.
32. Eiby YA, Booth DT. The effects of incubation temperature on the morphology and composition of Australian brush-turkey (*Alectura lathami*) chicks. J Comp Physiol B. 2009;179:875−882.
33. Koláčková M, Prokůpková L, Albrecht T, Hořák D. Incubation temperature influences trade-off between structural size and energy reserves in mallard hatchlings. Physiol Biochem Zool. 2015;88:1–10.
34. Gefen E, Ar A. Gas exchange and energy metabolism of the ostrich (*Struthio camelus*) embryo. Comp Biochem Physiol A. 2001;130:689–699.
35. Packard MJ, Packard GC. Water loss from eggs of domestic fowl and calcium status of hatchlings. J Comp Physiol B. 1993;163:327−331.
